# Selenium as an Antioxidant: Roles and Clinical Applications in Critically Ill and Trauma Patients: A Narrative Review

**DOI:** 10.3390/antiox14030294

**Published:** 2025-02-28

**Authors:** Jae-Gil Lee, Ji-Young Jang, Seung-Min Baik

**Affiliations:** 1 Department of Surgery, Ewha Womans University Mokdong Hospital, Seoul 07985, Republic of Korea; baiksm@ewha.ac.kr; 2 Department of Surgery, National Health Insurance Service Ilsan Hospital, Goyang 10444, Republic of Korea; jyjang@nhimc.or.kr

**Keywords:** selenium, antioxidants, critically ill, trauma, supplementation

## Abstract

Selenium plays an indispensable role in antioxidant defense through its incorporation into selenoproteins, including glutathione peroxidase (GPx) and thioredoxin reductase. In the context of trauma and critical illness, systemic inflammation and oxidative stress frequently deplete selenium reserves, compromising the body’s antioxidant defenses. This deficiency exacerbates immune dysfunction, elevates the risk of multi-organ dysfunction syndrome, and increases susceptibility to infections and mortality. Observational studies have consistently shown that lower selenium levels correlate with poorer clinical outcomes, such as extended stays in intensive care units and higher mortality rates. Supplementation of selenium has demonstrated promise in restoring GPx activity, reducing oxidative stress markers, and supporting recovery, particularly in patients with pre-existing selenium deficiency. While the impact on mortality remains variable across clinical trials, early and targeted supplementation appears to be beneficial, especially when combined with other micronutrients like vitamins C and E or zinc. These combinations enhance the antioxidant response and tackle the complex oxidative pathways in critically ill and trauma patients. Importantly, the clinical benefits of selenium supplementation appear to be influenced by baseline selenium status, with patients exhibiting severe deficiency deriving the most pronounced improvements in oxidative stress markers, immune function, and recovery. This review highlights the critical importance of addressing selenium deficiency, advocating for personalized therapeutic strategies. However, further large-scale studies are essential to optimize dosing regimens, refine combination therapies, and validate selenium’s therapeutic potential in trauma and critical care settings.

## 1. Introduction

When the body encounters infection or trauma, it initiates a cascade of inflammatory responses. What begins as a localized reaction to injury can rapidly escalate into a systemic response, marked by the release of cytokines and neurohormonal mediators. Alongside this, reactive oxygen species (ROS) and reactive nitrogen species (RNS) are generated as part of the immune defense. While these molecules play critical roles in fighting pathogens, their excessive accumulation often leads to oxidative stress, amplifying tissue damage [1,2,3]. To counteract this, the body activates its antioxidant defense mechanisms, which rely heavily on key micronutrients like selenium and other trace elements.

Vitamins and trace elements serve as indispensable cofactors or antioxidants, neutralizing ROS and maintaining cellular equilibrium. Selenium is central to mitigating oxidative stress and supporting immune function. However, critically ill patients often experience severe selenium depletion, attributed to multiple factors, including increased metabolic demand, impaired gastrointestinal absorption, and enhanced utilization during inflammatory and oxidative stress states [1,2,3]. Studies revealed that serum selenium levels can drop by 40–60% within 24 h of intensive care unit (ICU) admission [4,5,6], contributing to increased oxidative damage and poorer clinical outcomes, including increased infection rates, increased ventilator-associated pneumonia (VAP), prolonged ICU stays, and higher mortality [4,6,7,8,9,10,11]. Although selenium supplementation has been proposed as a strategy to restore antioxidant capacity, its effects in critically ill populations remain inconsistent across studies [12,13,14,15,16]. These inconsistencies are influenced by multiple factors, including patient heterogeneity, variations in dosing regimens, timing of administration, and baseline selenium levels. A more detailed discussion of these influencing factors will be provided later in the manuscript.

Guidelines for micronutrients from the American Society of Parenteral and Enteral Nutrition (ASPEN) and European Society for Clinical Nutrition and Metabolism (ESPEN) recommend providing essential micronutrients through parenteral nutrition to support metabolic processes and prevent deficiencies in critically ill patients [17,18,19,20]. However, routine high-dose antioxidant therapy is not universally endorsed, as highlighted by the 2016 ASPEN-SCCM and 2019 ESPEN critical care nutrition guidelines, which caution against such interventions unless specific deficiencies are documented emphasizing the need for personalized approaches based on patient-specific needs [17,18]. This reflects a cautious approach to additional supplementation in this vulnerable population.

This review explores the physiological changes associated with critical illness and trauma, focusing on the role of selenium in antioxidant defense. It explores its clinical relevance, as well as the benefits and limitations of selenium supplementation, in improving outcomes for critically ill and trauma patients.

## 2. Physiological Changes and Oxidative Responses in Critically Ill and Trauma Patients

**Figure 1:**
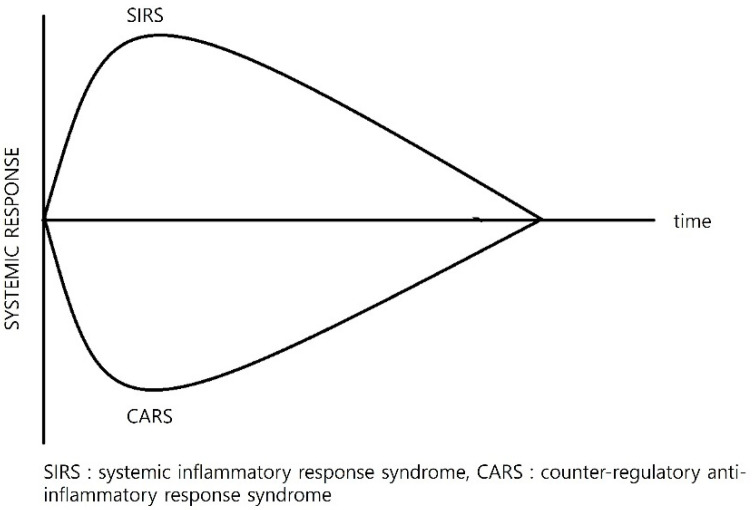
A simplified graph illustrating the systemic immune response following trauma, showing the progression of SIRS (systemic inflammatory response syndrome) and CARS (compensatory anti-inflammatory response syndrome) over time. The graph highlights how the body transitions from an initial inflammatory phase (SIRS) to an anti-inflammatory recovery phase (CARS), eventually returning to homeostasis if recovery is successful. SIRS typically emerges within the first few hours post-injury, driven by the release of pro-inflammatory cytokines such as IL-6, TNF-α, and IL-1β. CARS, characterized by anti-inflammatory mediators, follows within 24–48 h and can persist for several days to weeks, depending on the severity of trauma and host immune response. Recent evidence suggests that these two phases can overlap, with simultaneous pro- and anti-inflammatory signaling contributing to immune dysregulation in critically ill patients (Adapted from Cavaillon JM J, et al. J Endotoxin Res. 2001; 7 (2): 85–93. PMID: 11521088 [23]).

Critically ill and trauma patients often develop systemic inflammatory response syndrome (SIRS), which can escalate into multi-organ dysfunction syndrome (MODS) in severe cases [21,22]. The interplay between SIRS and compensatory anti-inflammatory response syndrome (CARS) creates a complex inflammatory environment. While these processes were once thought to occur sequentially, emerging evidence suggests that SIRS and CARS overlap, particularly in the early stages of injury [1,2,3] (Figure 1). SIRS is driven by the innate immune system, leading to the release of pro-inflammatory cytokines such as IL-6, TNF-α, and IL-1β [23]. These cytokines amplify inflammation, exacerbating tissue damage. On the other hand, CARS acts as a countermeasure, suppressing adaptive immune responses to mitigate inflammation. Although this anti-inflammatory mechanism can facilitate recovery, excessive immune suppression may result in immune paralysis, leaving patients vulnerable to infections, delayed wound healing, and late-stage organ failure [21,24]. In trauma patients, systemic inflammation is also fueled by damage-associated molecular patterns (DAMPs), such as HMGB1, mitochondrial DNA, and heat-shock proteins. These molecules mimic pathogen-associated molecular patterns (PAMPs), activating Toll-like receptors (TLRs) and other pattern recognition receptors. This triggers widespread inflammation, with trauma-induced mitochondrial DAMPs further intensifying oxidative stress and contributing to a sepsis-like state [22,25,26,27].

Ischemia-reperfusion injury (IRI) represents another significant challenge in critically ill and trauma patients. During ischemia, reduced oxygen supply impairs ATP production, disrupts cellular integrity, and causes ionic imbalances, leading to cellular damage. When blood flow is restored, oxygen reintroduction produces superoxide anions, which are converted into hydrogen peroxide (H_2_O_2_) by superoxide dismutase (SOD). These molecules undergo subsequent reactions to form highly reactive hydroxyl radicals and cytotoxic ROS. This process exacerbates lipid peroxidation, apoptosis, and oxidative tissue damage [28,29,30,31,32,33]. Recent studies highlight that IRI not only amplifies oxidative stress but also severely depletes antioxidant reserves in the early phase of trauma and sepsis, further increasing susceptibility to organ dysfunction [29,30]. Specifically, selenium-dependent glutathione peroxidase (GPx) and thioredoxin reductase (TrxR) activities are significantly reduced following reperfusion, impairing the body’s ability to neutralize ROS and regenerate key antioxidants such as glutathione [29]. Moreover, the severity of IRI has been linked to deficiencies in trace elements, including selenium, zinc, and copper, which are critical for enzymatic antioxidant defense mechanisms [30].

Oxidative stress, defined as an imbalance between ROS production and antioxidant defenses, is a hallmark of critical illness and trauma. Excessive ROS, generated during oxidative phosphorylation, ischemia-reperfusion injury, and systemic inflammation cause extensive damage to lipids, proteins, and DNA. This oxidative damage worsens mitochondrial dysfunction, disrupts immune regulation, and leads to organ injury. Furthermore, this creates a vicious cycle, as pro-inflammatory cytokines (e.g., IL-6, TNF-α) are released in response to DAMPs, perpetuating the inflammatory process [22,25,26,27,28,29,30].

Under normal conditions, the body’s antioxidant defense system comprising enzymatic antioxidants like glutathione peroxidase and SOD, along with non-enzymatic antioxidants such as selenium and vitamins C and E neutralizes ROS to maintain cellular balance [34]. However, in critically ill states, these defenses are often overwhelmed, resulting in uncontrolled oxidative stress. This imbalance contributes to complications such as MODS, delayed wound healing, cardiovascular issues, and neurological impairment. To counteract oxidative stress, therapeutic interventions have shown promise. High-dose vitamin C, N-acetylcysteine (NAC), and selenium supplementation have been explored for their ability to enhance antioxidant defenses and mitigate oxidative damage. These therapies not only target ROS accumulation but also reduce inflammatory cytokine production and support mitochondrial function. For example, selenium acts as a cofactor for glutathione peroxidase, a key enzyme in ROS detoxification, while vitamin C and NAC provide complementary antioxidant effects [10,35,36,37,38]. Despite these promising findings, clinical outcomes remain inconsistent, emphasizing the importance of personalized antioxidant strategies. Tailoring interventions to individual patient characteristics, timing, and dosage is essential for maximizing efficacy. Understanding the mechanisms driving oxidative stress and inflammation in critically ill and trauma patients will be critical for developing targeted therapies that minimize complications, preserve organ function, and improve survival outcomes in this high-risk population.

## 3. Physiological and Clinical Functions of Antioxidants

Antioxidants play an essential role in counteracting oxidative stress, a primary contributor to organ dysfunction and poor outcomes in critically ill and trauma patients. Oxidative stress arises from an imbalance between the production of ROS and the body’s antioxidant defenses. This imbalance is further aggravated by hyperinflammation, mitochondrial dysfunction, and immune impairment commonly observed in these conditions [4,39]. Micronutrients, such as selenium, zinc, manganese, copper, and vitamins C and E, are integral to the function of enzymatic antioxidants, including SOD, catalase, and GPx. These antioxidants work together to neutralize ROS and maintain cellular homeostasis [40,41,42,43,44,45,46]. For example, zinc plays a critical role in wound healing, immune modulation, and stabilization of glutathione levels. Zinc deficiencies, on the other hand, are linked to increased infection rates and worse clinical outcomes [47,48,49,50,51,52]. Similarly, manganese and copper are vital for mitochondrial and cytosolic SOD activity, and their deficiencies exacerbate oxidative stress and impair immune function [34,52,53,54]. Selenium, a key cofactor for GPx, helps reduce hydrogen peroxide and lipid hydroperoxides, protecting cells from oxidative damage. However, selenium deficiency—a common issue in critically ill patients—compromises mitochondrial function and increases vulnerability to MODS [46,48,55]. Notably, micronutrient deficiencies often coexist, particularly in critically ill patients with prolonged catabolic states, malnutrition, or critical organ dysfunction. Low zinc and selenium levels have been observed simultaneously in sepsis and trauma patients, further impairing antioxidant defenses and immune function. The presence of multiple deficiencies exacerbates oxidative stress, increasing the likelihood of organ failure and infectious complications [6,7,10,56,57].

Vitamins C and E also serve as potent antioxidants with distinct but complementary roles. Vitamin C helps modulate cytokine storms, enhances immune defense, and supports tissue repair, while vitamin E protects against lipid peroxidation and maintains the integrity of cellular membranes. Under stress conditions, such as trauma or critical illness, damaged mitochondria generate excessive ROS, creating a self-perpetuating cycle of oxidative stress and organ dysfunction [36,42,51,55,58,59,60,61,62]. Given the complexity of oxidative pathways, relying on a single antioxidant is often insufficient. Recent evidence underscores the effectiveness of combining multiple antioxidants to optimize recovery. For instance, selenium used alongside zinc, vitamins C and E, and other cofactors has demonstrated improved efficacy in mitigating oxidative stress and enhancing clinical outcomes [61,62]. The synergistic action of these antioxidants enhances redox balance, with vitamin C regenerating oxidized vitamin E, zinc- and copper-supporting SOD activity, and selenium-dependent GPx-neutralizing peroxides. This combined effect strengthens cellular antioxidant defenses, reduces inflammatory responses, and supports mitochondrial function, which is crucial for critically ill patients (Table 1). Therapeutic approaches, including high-dose vitamin C, NAC, and selenium supplementation, have shown promise in reducing oxidative damage and inflammation. However, clinical outcomes remain variable, highlighting the importance of personalized strategies. These strategies should account for patient-specific factors, such as baseline micronutrient deficiencies, timing of intervention, and appropriate dosing [37,38,40].

Several clinical trials have investigated multi-micronutrient supplementation strategies in both trauma and general ICU populations. Collier et al. reported that high-dose antioxidant therapy, including selenium, reduced ICU length of stay and mortality in trauma patients receiving enteral nutrition [38]. However, large-scale randomized controlled trials (RCTs) in mixed ICU populations, such as the SIGNET and REDOX trials, have shown inconsistent effects on mortality and infection rates, emphasizing the need for further research on patient-specific supplementation strategies [63,64]. By doing so, it is possible to improve clinical outcomes and promote recovery in critically ill and trauma patients.

### Roles of Selenium in Antioxidation

**Figure 2:**
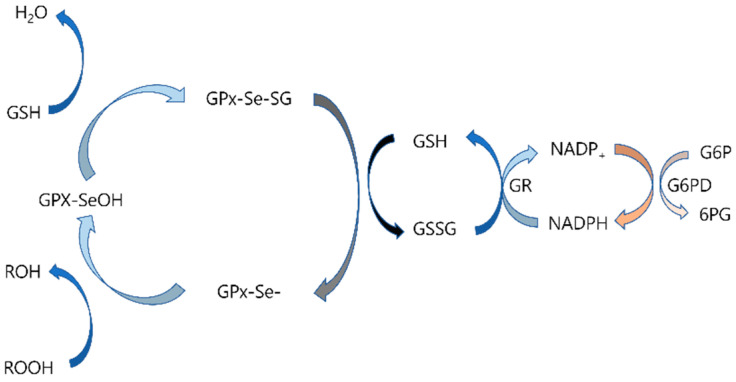
Mechanism of glutathione peroxidase (GPx) redox pathway. Selenol (-SeH) in GPx is oxidized to selenic acid (-SeOH) by peroxide (ROOH), releasing H_2_O. Glutathione (GSH) reduces -SeOH to a selenenyl-sulfide intermediate (-Se-SG), which is further reduced to regenerate selenol (-SeH) while forming oxidized glutathione (GSSG). GSSG is reduced back to GSH by NADPH-dependent glutathione reductase (GR), with NADPH regenerated via glucose 6-phosphate dehydrogenase (G6PD) (Adapted from Pei J, et al. Front Pharmacol. 2023. DOI:10.3389/fphar.2023.1147414 [66]).

Selenium is a crucial component of antioxidation, through incorporating into selenoproteins such as GPx. GPx catalyzes the conversion of H_2_O_2_ and lipid hydroperoxides into harmless byproducts, such as water and alcohols, using reduced glutathione (GSH) as an electron donor. This reaction prevents the accumulation of ROS, especially during periods of heightened oxidative stress, such as trauma or critical illness, and reduces lipid peroxidation, thus preserving the integrity of cellular membranes [9,11,65,66] (Figure 2).

The efficiency of GPx is directly influenced by selenium levels. In selenium-sufficient conditions, GPx exhibits a high catalytic efficiency, with a turnover number (kcat) of approximately 2000–5000 s^−1^, depending on the isoform and substrate [67,68]. However, selenium deficiency significantly reduces GPx activity, with studies reporting a 30–50% drop in enzymatic function, particularly in critically ill patients [67,68,69]. This reduction leads to increased ROS accumulation, lipid peroxidation, and mitochondrial dysfunction, exacerbating inflammation and oxidative injury.

In addition to its role in GPx, selenium is indispensable for the activity of thioredoxin reductase (TrxR), an enzyme that regenerates thioredoxin (Trx). The Trx/TrxR system is crucial for maintaining cellular redox balance by reducing oxidized proteins and protecting cells from oxidative damage. Beyond its antioxidative function, this system also influences intracellular signaling pathways that regulate inflammation and apoptosis, further lowering ROS levels and supporting mitochondrial health under stress conditions [11,67]. To provide a clearer understanding of the roles and clinical implications of selenoproteins, a summary table has been included (Table 2).

In vitro and animal studies have demonstrated that selenium inhibits the activation of the NLRP3 inflammasome, a key driver of inflammation, thereby reducing the production of pro-inflammatory cytokines such as IL-1β and IL-18 [69,70,71]. Additionally, preclinical models and human studies suggest that selenium suppresses the nuclear factor kappa B (NF-κB) pathway, which controls the expression of inflammatory mediators during oxidative and immune responses [12,26,65]. Through these mechanisms, selenium effectively curtails inflammation-induced oxidative stress, which is particularly detrimental in critically ill patients [8,9].

Since mitochondria are the primary sites of ROS production during oxidative phosphorylation, they are highly vulnerable to oxidative damage. Both in vitro and animal models indicate that selenium enhances GPx and TrxR activity within mitochondria, neutralizing ROS and preventing lipid peroxidation of mitochondrial membranes [26,65]. This preserves mitochondrial function and reduces the release of DAMPs, which would otherwise exacerbate inflammation and cellular injury. Human observational studies have further linked lower selenium levels with increased oxidative damage and mitochondrial dysfunction in critically ill patients [8,9,21,69,70,71]. Selenoprotein P (SELENOP) provides additional antioxidative support by functioning as both a selenium transporter and an extracellular scavenger of ROS. By delivering selenium to tissues and protecting endothelial cells from oxidative damage, SELENOP promotes vascular health and mitigates systemic oxidative stress [9,21,48].

Selenium’s ability to modulate redox-sensitive signaling pathways is also crucial for regulating apoptosis and enhancing cellular survival during oxidative stress. By reducing ROS levels and limiting oxidative damage, selenium strengthens cellular resilience, particularly under high-stress conditions such as trauma, sepsis, and ischemia-reperfusion injury [21,48,71]. Collectively, these antioxidative mechanisms underscore selenium’s critical role in protecting cells and tissues from oxidative damage. Selenium supplementation has demonstrated the ability to restore antioxidant enzyme activity, reduce inflammation, and support mitochondrial function, ultimately improving outcomes in critically ill and trauma patients. By enhancing GPx and TrxR activity, modulating inflammatory pathways, and preserving mitochondrial integrity, selenium provides significant therapeutic potential for managing conditions characterized by excessive ROS production.

## 4. Methodology

A comprehensive literature search was conducted using PubMed to identify studies on selenium in critically ill and trauma patients, focusing on two main areas: selenium deficiency, and selenium supplementation. The search included keywords such as “selenium” or “selenoprotein” or “selenium supplementation” or “selenite” and (“critically ill” or “ICU” or “trauma”), with no date restrictions to encompass both historical and recent research. Studies were selected based on predefined inclusion and exclusion criteria to ensure relevance to the research objectives. Studies were included if they investigated patients aged 18 years or older who were critically ill and receiving ICU treatment or those who had sustained trauma or burns requiring treatment. Only studies that analyzed selenium levels or examined selenium supplementation as part of the intervention were considered. Studies involving pediatric populations were excluded, as well as those that did not specifically analyze selenium or include selenium supplementation in their methodology.

To ensure a broad and comprehensive analysis, studies included in this review were not limited to randomized controlled trials (RCTs) but also encompassed observational studies, cohort studies, and prospective trials that investigated selenium deficiency, selenium concentrations, or selenium supplementation in critically ill and trauma patients. Research with varied dosing regimens, intervention durations, and selenium formulations was incorporated to capture the diversity of selenium administration in clinical practice, ensuring a comprehensive evaluation of its clinical implications. Furthermore, research examining selenium’s interactions with other antioxidants, including vitamins C and E, zinc, and copper, was included to provide a mechanistic understanding of its antioxidative effects.

Since this is a narrative review, no formal meta-analysis was conducted; instead, findings from selected studies were synthesized to highlight key clinical outcomes, such as mortality, ICU length of stay, inflammatory markers, and oxidative stress levels. The selection process prioritized studies with clinical relevance, ensuring that high-quality observational and interventional studies were incorporated to provide a comprehensive understanding of selenium’s role in critical illness and trauma.

## 5. Selenium in Critically Ill Patients

Selenium, an essential trace element, is fundamental to human health, primarily through its role in selenoproteins like GPx and TrxR. These enzymes maintain redox balance, neutralize ROS, and regulate inflammation. Selenium exists in organic forms (selenocysteine, selenomethionine) and inorganic forms (selenite, selenate), with a daily requirement of 55–70 µg for adults [19,20,40].

Critically ill patients often experience selenium depletion due to oxidative stress, systemic inflammation, and increased metabolic demands. Reduced gastrointestinal absorption and increased utilization during acute stress states exacerbate this deficiency. Studies consistently show plasma selenium levels in critically ill patients can decrease by up to 40–60% compared to healthy persons [4,6,57,72,73]. This depletion highlights the need for selenium supplementation to restore antioxidant defenses and mitigate oxidative stress. Selenium deficiency has been linked to increased oxidative stress and organ dysfunction. Reduced GPx activity compromises the detoxification of ROS, leading to lipid peroxidation, protein damage, and DNA injury, which can accelerate MODS. Patients with lower selenium levels exhibit higher incidences of acute respiratory distress syndrome (ARDS), prolonged mechanical ventilation, and acute kidney injury (AKI) [4,6,11]. Selenium’s role in reducing oxidative damage is crucial for maintaining cellular homeostasis during critical illness. The immune system also suffers from selenium deficiency. Impaired neutrophil and macrophage function weakens the body’s defense against infections. Low selenium levels correlate with increased ventilator-associated pneumonia (VAP), bloodstream infections, and sepsis-related complications, prolonging recovery and raising the risk of secondary infections [7,8,10]. Observational studies have identified a strong association between low plasma selenium levels and higher mortality rates in ICU patients. Patients in the lowest quartile of selenium levels have a significantly increased risk of 28-day mortality compared to those with adequate levels. Furthermore, selenium deficiency has been shown to predict prolonged ICU stays, higher infection rates, and increased hospital costs, underscoring its clinical and economic implications in critical care [4,6,11,52]. In patients with sepsis and septic shock, selenium deficiency is linked to elevated pro-inflammatory cytokines like TNF-α, IL-1β, and IL-6, exacerbating systemic inflammation and worsening outcomes [5,49,74]. Early identification and targeted intervention for selenium deficiency are critical for improving clinical outcomes. Table 3 further details specific studies that illustrate the impact of selenium deficiency on critical illness, highlighting key findings such as correlations with inflammatory markers, oxidative damage, and clinical severity.

Selenium supplementation has shown promise in restoring antioxidant activity, reducing inflammation, and supporting immune function. Numerous studies underscore its potential to improve clinical outcomes (Table 4). For instance, the SIGNET trial demonstrated that selenium supplementation at 500 µg/day enhanced antioxidant defenses in patients receiving parenteral nutrition, though it did not significantly impact mortality or infection rates. Prolonged supplementation (≥5 days) was associated with a reduction in new infections, suggesting that timing and duration may influence outcomes. High-dose selenium has been shown to improve antioxidant reserves and reduce biomarkers of oxidative stress in patients with severe sepsis, trauma, and burns. Valenta et al. found that selenium increased GPx activity and reduced inflammatory markers like IL-6 and CRP, though no significant effect on 28-day mortality was observed [16]. Similar benefits were reported in mechanically ventilated patients, with reductions in oxidative stress biomarkers but no significant decrease in VAP incidence [12,14]. Meta-analyses offer broader insights into selenium’s efficacy. A 2015 meta-analysis of 16 randomized controlled trials (RCTs) found selenium supplementation reduced overall mortality, particularly with sodium selenite. However, secondary outcomes, such as ICU length of stay and mechanical ventilation duration, showed inconsistent results [35]. Conversely, a 2016 meta-analysis concluded selenium had no significant impact on mortality, regardless of the dosing strategy [15]. Importantly, these meta-analyses did not always account for baseline selenium status, making it difficult to determine whether supplementation was more beneficial for patients with existing selenium deficiency. Additionally, some studies included different selenium formulations (e.g., sodium selenite vs. organic selenium compounds), which may influence bioavailability and efficacy. A 2019 trial sequential analysis suggested selenium monotherapy could reduce mortality and hospital stays in patients with severe oxidative stress or baseline selenium deficiency [16]. Despite these biochemical benefits, clinical outcomes of selenium supplementation remain variable, influenced by factors such as baseline selenium status, formulation, dosing strategies, timing of intervention, and patient heterogeneity. The inconsistency in mortality outcomes across meta-analyses on selenium supplementation in critically ill patients is largely due to heterogeneity in study designs, patient populations, dosing regimens, and baseline selenium status. Variability in selenium dosing (ranging from 300 to 1000 µg/day), timing of administration, and the failure to stratify patients based on baseline selenium levels contribute to these mixed findings. Additionally, the lack of standardized deficiency thresholds and biomarker-driven patient selection may have diluted potential benefits. While high-dose selenium shows potential in patients with severe oxidative stress, caution is needed for individuals with renal dysfunction or those undergoing renal replacement therapy, as excessive supplementation may pose toxicity risks [64,75].

**Figure 3:**
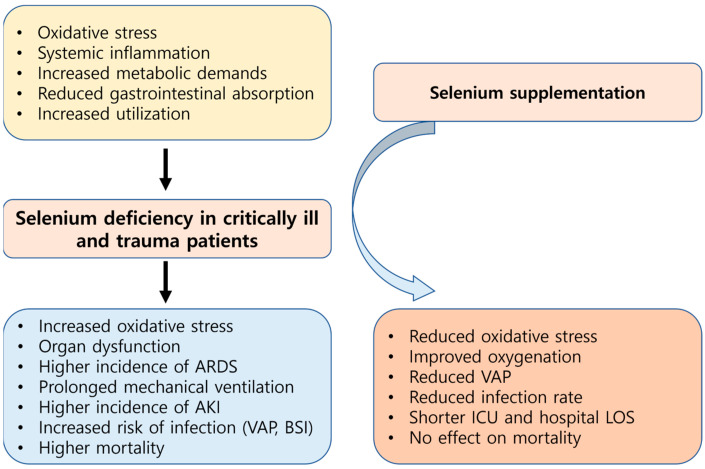
Conceptual summary of clinical outcomes related to selenium deficiency and selenium supplementation in critically ill and trauma patients. This figure outlines the causes of selenium deficiency, including oxidative stress and inflammation, as well as the complications such as ARDS, AKI, and infections like VAP and BSI. It also illustrates the potential benefits of selenium supplementation, including reduced oxidative stress, improved oxygenation, and shorter ICU stays. This figure is a conceptual representation summarizing key findings from multiple studies rather than data from a single source. ARDS, acute respiratory distress syndrome; AKI, acute kidney injury; VAP, ventilator-associated pneumonia; BSI, bloodstream infection, ICU, intensive care unit; LOS, length of stay.

The causes of selenium deficiency, complications (or outcomes) resulting from deficiency, and the effects of selenium supplementation in critically ill patients are summarized in Figure 3. The illustration highlights the mechanisms of selenium deficiency driven by oxidative stress and systemic inflammatory responses in critically ill and trauma patients, as well as the associated clinical outcomes. It also demonstrates the beneficial effects of selenium supplementation, emphasizing the clinical importance of selenium and supporting the need for personalized micronutrient supplementation strategies in critically ill care.

## 6. Selenium in Trauma Patients

Trauma patients are particularly vulnerable to selenium deficiency due to the systemic inflammatory response, oxidative stress, and metabolic shifts that occur during acute injury. Simultaneous activation of SIRS and CARS drives excessive oxidative stress, mitochondrial dysfunction, and cellular damage. Observational studies have consistently reported that plasma selenium levels and selenoprotein P concentrations drop sharply within the first 1–6 h after severe trauma and often remain low for up to 72 h, especially in non-survivors [11,70,71]. This depletion reflects increased selenium utilization during heightened oxidative stress and inflammation, compounded by impaired absorption in critically ill states.

The clinical implications of selenium deficiency in trauma are significant. As a critical cofactor for GPx, selenium depletion leads to reduced GPx activity, resulting in ROS accumulation, lipid peroxidation, and DNA damage. These changes exacerbate mitochondrial dysfunction, impair immune responses, and increase the risk of MODS and mortality. For example, Choi et al. reported that trauma patients with selenium deficiency within 48 h of ICU admission had significantly higher 30-day mortality rates and increased incidences of pneumonia and other infectious complications [7]. Persistent selenium depletion has also been linked to prolonged ICU stays, elevated Sequential Organ Failure Assessment (SOFA) scores, and overall poorer outcomes, highlighting selenium’s critical role in trauma recovery and survival (Table 5).

Selenium supplementation has been investigated as a potential strategy to counteract these effects (Table 6). Early supplementation has shown promise in restoring antioxidant defenses and reducing complications. For instance, a prospective observational study on traumatic brain injury (TBI) found that selenium supplementation significantly reduced unfavorable functional outcomes at discharge and follow-up (adjusted RR: 0.61; 95% CI: 0.44–0.83; *p* = 0.002) [81]. Similarly, trace element supplementation in burn patients, including selenium, improved plasma antioxidant levels, reduced pneumonia and wound infections, and enhanced tissue healing [52]. These findings underscore selenium’s role in supporting the antioxidant network and mitigating the effects of severe oxidative stress. However, studies specifically focused on trauma-related conditions remain limited. Moderate selenium doses (300–800 µg/day) have shown success in improving outcomes, while high-dose regimens (e.g., 3000–4000 µg/day) have produced inconsistent results, with limited impact on mortality or ICU length of stay [15,35]. Emerging evidence suggests that selenium supplementation alone may not be sufficient to fully restore antioxidant defenses in trauma patients. Selenium works synergistically with other micronutrients, including vitamin C, vitamin E, zinc, copper, and manganese, which together play complementary roles in reducing oxidative stress and supporting immune function [40,45]. Combination supplementation strategies have demonstrated enhanced efficacy, particularly in patients with severe oxidative stress and significant baseline deficiencies. For instance, Berger et al. conducted a study in major burn patients, demonstrating that a combination of selenium, vitamin C, and zinc improved wound healing and reduced infection rates [52,82]. Another trial in trauma patients receiving enteral nutrition with a combination of selenium, vitamin C, and vitamin E reported improved antioxidant capacity and reduced inflammatory markers [62]. Additionally, Collier et al. evaluated the impact of high-dose antioxidant supplementation, including selenium, in acutely injured patients. Their findings demonstrated that antioxidant therapy was associated with significantly shorter hospital and ICU stays, as well as a reduction in mortality (OR, 0.32; 95% CI, 0.22–0.46), after adjusting for injury severity [38]. These findings suggest that a multimodal antioxidant approach may be more beneficial than selenium monotherapy in trauma-related conditions.

To improve clinical outcomes for trauma patients, future research should prioritize standardizing selenium supplementation protocols, defining optimal dosing regimens, and evaluating the benefits of combination therapies. Reliable biomarkers, such as selenoprotein P, are also needed to predict outcomes, monitor supplementation effects, and tailor interventions to individual patient needs. High-quality, large-scale randomized controlled trials (RCTs) will be essential for establishing definitive evidence of selenium’s role in trauma care.

## 7. Discussion

### 7.1. Critical Appraisal of Clinical Evidence

While numerous studies have investigated the effects of selenium supplementation in critically ill and trauma patients, significant methodological variations exist, contributing to inconsistent clinical outcomes. One major factor is heterogeneity in patient populations, as studies include diverse cohorts such as trauma patients, sepsis patients, ARDS patients, burn patients, and general ICU populations, each with varying degrees of oxidative stress and baseline selenium levels. Additionally, differences in selenium formulations (organic vs. inorganic selenium, sodium selenite vs. selenomethionine), dosing regimens (low-dose vs. high-dose selenium, short-term vs. prolonged administration), timing of supplementation (early vs. delayed administration), and route of supplementation (enteral vs. parenteral) further complicate comparisons across trials. Meta-analyses examining selenium supplementation have also yielded conflicting conclusions, with some demonstrating a mortality benefit and others showing no significant effect [15,16]. This variability may stem from differences in study inclusion criteria, endpoint selection (e.g., mortality, ICU length of stay, inflammatory markers), and the failure to stratify patients based on baseline selenium status. Many studies do not account for pre-existing selenium deficiency, which could influence responsiveness to supplementation. Furthermore, some trials lack blinding or placebo control, increasing the risk of bias. Given these inconsistencies, future research should focus on well-designed, large-scale RCTs that incorporate biomarker-guided stratification (e.g., SELENOP levels, GPx activity) to identify subgroups that would benefit most from selenium supplementation. Standardized protocols for selenium dosing and administration are also necessary to enhance reproducibility and establish clear clinical guidelines.

### 7.2. Inconsistencies in Clinical Outcomes

Despite strong mechanistic evidence supporting the role of selenium in oxidative stress modulation and immune function, clinical trials evaluating selenium supplementation in critically ill and trauma patients have produced inconsistent outcomes. Several key factors contribute to this variability, including differences in baseline selenium status, dosing strategies, timing of administration, study design, and patient heterogeneity.

#### 7.2.1. Baseline Selenium Status and Deficiency Severity

One major source of inconsistency is the baseline selenium status of patients included in clinical trials. Selenium deficiency is common in critically ill patients, but its severity varies significantly across populations and geographical regions. Some studies include patients with profound selenium depletion, who may benefit more from supplementation, while others involve patients with only mild deficiencies or normal selenium levels at baseline. Trials that fail to account for baseline selenium levels may underestimate the potential benefits of supplementation, as selenium-replete patients are unlikely to experience significant improvement.

#### 7.2.2. Variability in Dosing Strategies

The dosage and form of selenium supplementation differ widely among studies, making direct comparisons challenging. Studies have used doses ranging from 300 µg/day to 4000 µg/day, with some employing high loading doses followed by maintenance dosing, and others using a fixed daily regimen. Furthermore, different forms of selenium (e.g., sodium selenite, selenium, and enteral selenium) have distinct bioavailability and metabolic pathways, potentially leading to variations in efficacy. A standardized dosing protocol tailored to selenium-deficient patients is needed to improve consistency across trials.

#### 7.2.3. Timing of Supplementation

The timing of selenium administration is another crucial variable. Some studies initiate supplementation within the first 24 h of ICU admission, while others begin treatment later in the disease course. Given that oxidative stress and inflammatory damage peak early in critical illness and trauma, delayed selenium administration may limit its protective effects. Future studies should focus on early intervention, ideally within the first 6–12 h of ICU admission, to maximize its potential benefits.

#### 7.2.4. Study Design and Heterogeneity

Differences in study design and patient populations further contribute to inconsistent findings. Some trials have focused on heterogeneous ICU populations, including patients with sepsis, trauma, burns, or post-surgical complications, making it difficult to isolate selenium’s effects in specific conditions. Additionally, some studies have small sample sizes or lack sufficient power to detect meaningful clinical differences, while others fail to control for confounding factors such as concurrent antioxidant therapy, severity of illness, and pre-existing nutritional deficiencies. Future well-powered, multicenter randomized controlled trials (RCTs) with stratified patient enrollment based on selenium status are essential to provide clearer evidence.

#### 7.2.5. Combination Therapy vs. Monotherapy

Emerging evidence suggests that selenium supplementation alone may not be sufficient to fully mitigate oxidative stress in critically ill patients. Combination therapy with other antioxidants, such as vitamin C, vitamin E, and zinc, has shown greater efficacy in some trials by providing complementary protection against oxidative damage and inflammation. However, studies differ in whether selenium was administered alone or as part of a multi-micronutrient approach, leading to discrepancies in reported benefits. Future research should explore synergistic antioxidant strategies and identify optimal combinations for critically ill and trauma patients.

## 8. Conclusions and Future Perspectives

Selenium plays a pivotal role in mitigating oxidative stress, modulating inflammation, and enhancing immune function in critically ill and trauma patients. Its incorporation into essential selenoproteins, such as GPx and TrxR, is fundamental to neutralizing ROS and preserving cellular homeostasis. However, trauma and critical illness often lead to rapid selenium depletion, exacerbating oxidative damage, impairing immune defenses, and contributing to organ dysfunction. These deficiencies are associated with adverse clinical outcomes, including higher mortality rates, prolonged ICU stays, and increased susceptibility to infections.

Selenium supplementation has demonstrated potential in restoring antioxidant defenses, reducing oxidative stress, and improving recovery, particularly in patients with pre-existing deficiencies or elevated oxidative stress. While observational studies consistently link selenium deficiency to worse outcomes, randomized clinical trials have shown inconsistent results, especially concerning mortality reduction and ICU length of stay. These discrepancies underline the importance of adopting a personalized approach to selenium therapy, focusing on optimal dosing, timing, and synergy with other antioxidants, such as vitamin C, vitamin E, and zinc.

Future research should prioritize large-scale, well-designed clinical trials to establish standardized supplementation protocols, identify patient subgroups most likely to benefit, and validate selenium’s therapeutic efficacy in trauma and critical care settings. Determining optimal dosing strategies is critical, as studies have suggested that medium-dose selenium supplementation (300–800 µg/day) may be more effective than high-dose regimens (≥3000 µg/day), which have shown inconsistent mortality benefits. The timing of selenium administration also warrants further investigation, particularly in the early phase of critical illness or trauma, when oxidative stress peaks. The use of biomarkers such as selenoprotein P and GPx activity should be integrated into future trials to stratify patients based on selenium status and monitor treatment efficacy. These biomarkers could help personalize selenium supplementation strategies, ensuring that patients with the highest oxidative stress burden and the most profound deficiencies receive targeted intervention. Additionally, combination therapy strategies incorporating selenium with other micronutrients (e.g., vitamin C, vitamin E, zinc, and copper) should be explored. Investigating the interplay between selenium supplementation and other antioxidant therapies may lead to more effective and personalized treatment strategies for critically ill and trauma patients.

Ultimately, early recognition and comprehensive management of selenium deficiency hold significant promise for improving survival rates, reducing complications, and expediting recovery in patients facing critical illness and severe trauma. By addressing these gaps, selenium supplementation could become a cornerstone of personalized care in critical illness and trauma management.

## Figures and Tables

**Table 1:** Synergistic roles of selenium and other antioxidants in oxidative stress regulation.

Antioxidant	Primary Function	Interaction with Selenium	Clinical Implication
Vitamin C	Regenerates vitamin E, neutralizes ROS, supports immune function	Enhances selenium-dependent GPx activity by reducing oxidative intermediates	Reduces inflammation, improves endothelial function, supports immune responses
Vitamin E	Prevents lipid peroxidation, protects cell membranes	Works alongside GPx to reduce lipid hydroperoxides	Protects against oxidative damage in critically ill patients
Zinc	Cofactor for SOD, modulates immune function	Works with selenium to maintain cellular redox balance	Improves immune response, reduces infection risk
Copper	Essential for SOD activity, supports mitochondrial function	Enhances antioxidant response with selenium-containing enzymes	Reduces oxidative stress, prevents mitochondrial dysfunction
Manganese	Cofactor for mitochondrial SOD, regulates oxidative stress	Works with selenium to detoxify peroxides	Protects against mitochondrial injury and oxidative damage
Coenzyme Q10	Involved in mitochondrial electron transport chain, protects against oxidative damage	Works with selenium to prevent mitochondrial dysfunction	Enhances cellular energy production and reduces oxidative damage in trauma patients

ROS, radical oxygen species; SOD, superoxide dismutase; GPx, glutathione peroxidase.

**Table 2:** Summary of selenoproteins, their expression organs, primary functions, and clinical implications. Selenoproteins play essential roles in redox regulation, immune function, and cellular homeostasis. Their expression varies across tissues, influencing antioxidant defense, inflammatory response, and disease susceptibility.

Selenoprotein	Expression Organs	Function	Clinical Implications
Glutathione peroxidase (GPx)	Liver, kidney, heart	Neutralizes hydrogen peroxide (H_2_O_2_) and lipid peroxides	Reduces oxidative stress, protects cell membranes, decreases inflammatory damage
Thioredoxin reductase (TrxR)	Liver, Brain, Immune Cells	Regulates redox balance, regenerates antioxidants	Modulates immune response, prevents mitochondrial dysfunction
Selenoprotein P (SELENOP)	Liver, Plasma	Selenium transport, antioxidant defense	Low levels linked to increased mortality, infection risk, and poor recovery in ICU patients
Selenoprotein W (SelW)	Muscle, Brain	Protects muscle cells from oxidative damage	Potential role in preventing muscle wasting in critically ill patients
Selenoprotein S (SelS)	Endoplasmic Reticulum	Regulates inflammatory cytokine production	May influence systemic inflammatory response syndrome (SIRS)
Selenoprotein K (SELENOK)	Immune Cells, Heart	Regulates calcium flux and immune cell function	Plays a role in immune function, cardiac protection, and modulation of autoimmune responses

ICU, intensive care unit; SIRS, systemic inflammatory response syndrome.

**Table 3:** Summary of studies for selenium deficiency in critically ill patients.

Population	Study Design	Normal Range/Definition of Depletion	Measured Biomarkers	Key Findings	Limitation
Surgical patients with sepsis (n = 27) [5]	Single center, Prospective	Se: 95.0 to 165.0 ng/mL Zn: 66 to 110 µg/dL NA for depletion	Serum TAC, Se, Zn	No difference in outcome according to the serum Se, Total TAC is correlated with severity	Small number, no control group, no exogenous antioxidants
Sepsis (n = 39) [6]	Singer center, Prospective	Se: variable Zn: 10–17 µM. Se depletion: below 0.5 μM	Serum Se, Zn, oxidative markers	Lower Se level in sepsis, inflammatory markers were inversely related to serum level.	Small number
SICU > 3 days (n = 162) [10]	Single center, Retrospective observational	Se: 95.0 to 165.0 ng/mL Zn: 66 to 110 µg/dL NA for depletion	Serum Zn, Se	No difference in Se levels between surviors and non-survivors, low Se level in shock patents.	Retrospective, small number
Sepsis (n = 95) [57]	Single center, Observational (cross sectional)	Se: 0.9 to 2.0 µmol/L Zn: 12–18 µmol/L NA for depletion	Zn, Se	Mean serum Se was significantly lower in patients with greater severity	Difficult 24 h urine collection, no correction of Se for urinalry Cr.
Critically ill patients on CVVHD (n = 11) [72]	Single center, Prospective observational	Se: 0.8 to 1.6 µmol/L Zn: 12.7–20.2 µmol/L NA for depletion.	Cu, Zn, Se in plasma and effluent	Se losses occurred during CVVHD, contributing to deficiency in critically ill patients.	Small sample size, no control group
Sepsis (n = 87) [74]	Single center, Prospective observational	NA	Fe, Cu, Zn, Co, Cr, Se, V, Ni, Cd, Al	No differences in Se between sepsis and control. No effects on mortality	Small control group, no FU measurement of serum level

Se, selenium; Zn, zinc; TAC, total antioxidation capacity; NA, not addressed; Cr, creatinine; CVVHD, continuous venovenous hemodiafiltration; RCT, randomized controlled trial; SIRS, systemic inflammatory response syndrome; FU, follow-up, Fe, iron; Cu, cooper; Co, cobalt; Cr, chromium; V, vanadium; Ni, nickel; Cd, cadmium; Al, aluminum.

**Table 4:** Summary of studies for selenium supplementation in critically ill patients.

Population	Study Designs	Intervention	Outcome Variables	Results	Study Limitation
ARDS (n = 50) [12]	Single-center RCT	IV Na-Se vs. NS for 10 days	Oxidative stress markers, oxygenation, mortality, duration of MV, ICU stay	Reduced oxidative stress markers; improved oxygenation but no significant mortality, MV duration, ICU stay	Small sample size, single-center study, short follow-up duration
Severe septic (n = 40) [13]	Single-center RCT	IV 1000 µg Na-Se (max. 14 d) vs. Non Se	Oxidative stress markers, antioxidant balance	Improved oxidative stress balance, increased antioxidant enzyme activity,	Small sample size, no clinical outcome
Critically ill (n = 99) [14]	Single-center RCT	IV 3 mg Na-Se + 1.5 mg Na-Se (2–10 days) vs. NS	Serum GPx-3 activity, VAP, death, ICU stay, vasopressor requirement	Increased Se and GPx = 3 levels, no differences in VAP or death	Small sample size, short duration of study
MOF with MV (n = 1223) [63]	Multicenter RCT	IV glutamine vs. placebo, IV antioxidants (300 µg Se, 20 mg Zn, 10 mg β-carotene, 500 mg Vit E, 1500 mg Vit C) vs. placebo	28-day mortality, ICU days, infectious complications, MOD, duration of MV, etc.	No benefit for new infection or 6-month mortality, and other clinical outcomes	Possible insufficient dose of Se or ineffective dosing schedule, no baseline Se level
Critically ill (n = 502) [64]	Multicenter RCT	PN vs. PN + Glu vs. PN + 500 µg Se vs. PN + Glu + 500 µg Se	New infection, ICU days, day of antibiotics use, death	No significant mortality reduction; improved oxidative biomarkers.	Lack of individualized nutritional support, variability in timing of supplementation, short infection monitoring
SIRS, sepsis, septic shock (n = 150) [73]	Single-center RCT	IV Na-Se (1000 µg + 500 µg 5–14 days) vs. PN Se (<75 µg)	Plasma Se, inflammaotry markers, 28 d mortality	No difference in mortality, trend to reduced mortality in SIRS	Small SIRS patients
Sepsis (n = 150) [75]	Single-center RCT	IV Na-Se (1000 µg + 500 µg 5–14 days) vs. PN Se (<75 µg)	Inflammatory markers, 28 d mortality	Increased Se and GPx activity, similar mortality	Not blinded to clinician, long study periods, no subgroup analysis
Postoperative critically ill patients with severe sepsis (n = 1040) [76]	Retrospective	(EN 100 µg Se/d or PN 105 µg Na-Se) ± adjuvant IV 1000 µg Se for 14 days	In-hospital mortality	No significant impact on mortality	Retrospective study, no clear indications for Se supplementation, Se supplementation for more severe patients, no adjusted variables
Septic shock patients (n = 60) [77]	Multicenter RCT	IV Na-Se (4000 µg + 1000 µg for 9 days) vs. placebo	Vasopressor day, duration of MV, mortality	No difference in mortality	Small sample size
SIRS, sepsis, and septic shock patients (n = 249) [78]	Multicenter RCT	IV Na-Se (1000 µg for 14 days) vs. placebo	28-day mortality, survival time, clinical course, Se, GPx-3 level	Improved mortality (42.4% vs. 56.7%, *p* = 0.049), increase serum Se and GPx-3 activity	Unpowered study population
Severe sepsis or septic shock (n = 76) [79]	Single-center RCT	IV Na-Se (1000 µg for discharge) vs. placebo	Immune function, oxidative stress markers	No differences in immune response	small sample size, variability in enrollment timing, and the inability for detailed cell-specific immune analyses
Mechanically ventilated septic patients (n = 70) [80]	Single-center RCT	IV Na-Se (2000 µg + 1500 µg for 14 dyas) vs. placebo	28 d mortality, inflammatory markers, duration of MV, ICU stay, VAP	No differences in mortality, and inflammatory markers, decreased VAP	Small sample size, single-center study, single blind study

RCT, randomized controlled trial; IV, intravenous; Se, selenium; NS, normal saline; PN, parenteral nutrition; IL, interleukin; CRP, C reactive protein; FRAP, ferric reducing antioxidant power; MV, mechanical ventilation; VAP, ventilator-associated pneumonia; MOF, multiple organ failure; ARDS, acute respiratory distress syndrome; Na-Se, sodium selenite; ICU, intensive care unit; GPx, glutathione peroxidase; Zn, zinc; Vit, vitamin; MOD, multiple organ dysfuction; Glu, glutamine; SIRS, systemic inflammatory response syndrome.

**Table 5:** Summary of studies for selenium deficiency in trauma patients.

Population	Study Design	Normal Range/Definition of Depletion	Measured Biomarkers	Key Findings	Limitation
Multiple trauma (n = 135) [7]	Single center, Retrospective cohort	Se below <70 ng/mL	Serum Se	Se deficiency was associated with (1) Higher 30-day mortality (8.3% vs. 0%; *p* = 0.018), (2) Increased incidence of pneumonia (66.7% vs. 28.3%; *p* < 0.001), (3) Higher risk of infectious complications (88.3% vs. 46.5%, *p* < 0.001)	Retrospective, small patients numbers
Major blunt trauma (ISS ≥ 16) (n = 24) [8]	Single center, Prospective observational	NA	Serum Se and SELENOP	Lower initial Se and SELENOP who died	Small patients number, many compounding factors to affect the Se level
Traumatic spinal cord injury (n = 52) [54]	Single center, Prospective observational	Se: 46–143 µg/L NA for depletion	Serum Se, SELENOP, Cu, CP	Significantly decreased levels of Se, Cu, SELENOP, and Ceruloplasmin within 24 h in patients with remission of neurological impairment.	Small patients number

Se, selenium; NA, not addressed; ISS, injury severity score; SELENOP, selenoprotein P; Cu, cooper; CP, ceruloplasmin.

**Table 6:** Summary of studies for selenium supplementation in trauma patients.

Population	Study Designs	Intervention	Outcome Variables	Results	Study Limitation
Major trauma, CS, SAH n = 200 [37]	Single-center RCT	AOX (IV Se 540 µg + Zn 60 mg + Vit C 2700 mg + others) vs. placebo for 5 days	Change in AKI score, infectious complications, ICU stay, mortality	No differences in clinical outcomes Reduced inflammatory response in trauma patients	Underpowered, lacked post-ICU SOFA score data, heterogeneity patients
Trauma ICU (n = 4294) [38]	Prospective observational	AO (IV AA 1000 mg + AT 1000 IU EN (3 times) + IV Se 200 µg) vs. non-AOX	Mortality, ICU days, MV days	Lower mortality (6.1 vs. 8.5%, *p* = 0.001) Shorter ICU LOS	Non RCT
Major burns (TBSA > 20%), n = 21 [39]	Single-center RCT	IV AOX(Se 4.8 μmol + Zn 574 μmol + Cu 59 μmol) + IV multivitamin for 14–21 days vs. none + IV multivitamin	Skin GPx, GR, glutathione	Increased skin tissue content Improved wound healing	Small number
Traumatic brain injury (n = 307) [81]	Prospective Quasi-RCT	IV Se 1000 µg for 5 days + 500 µg for 5 days vs. None	Mortality, ICU stay.	No survival benefit Significant reduction in the risk of unfavorable functional outcomes (GOS-E ≤ 4)	Not formally randomized
Major burns (TBSA > 30%), n = 20 [82]	Single-center RCT	IV Standard TE + IV AOX (Se 2.93 μmol + Zinc 402 μmol + Cu 42 μmol) vs. IV standard TE	LOS, infectious complications, respiratory complications	Reduction in infectious complications (1.9 vs. 3.1, *p* < 0.05), shorter hospital stays	Small patient number
Major trauma (mean ISS = 30) (n = 31) [83]	Single-center RCT	IV Se 500 µg (±AT 150 mg, Zn 13 mg) for 5 days vs. placebo	Hormones	Earlier normalization of thyroid hormones	Small patient number, no consideration for prior thyroid function

CS, cardiac surgery; SAH, subarachnoid hemorrhage; AOX, antioxidants; Se, selenium, Zn, zinc; AA, ascorbic acid; AT, alpha-tocopherol; EN, enteral; ICU, intensive care unit; LOS, length of stay; TBSA, total body surface area; Cu, copper; GPx, glutathione peroxidase; GR, glutathione reductase; GOS-E, Glasgow outcome score extended; TE, trace elements; ISS, injury severity score; SOFA, Sequential Organ Failure Assessment.
